# Progress in Disease of Digestive Organs

**Published:** 1901-12-14

**Authors:** 


### Progress in Disease of Digestive Organs.

**Carcinoma of Stomach.**—Most of the signs which have been put forward as indicative of this disease have been shown to be unreliable. The absence in it of leucocytosis during digestion has been held as doubtful as the rest, and, indeed, this phenomenon has been found, though less frequently in chronic gastric catarrh and ulcer. Carstairs Douglas[1] examined 11 cases of proved carcinoma, and found that this leucocytosis, though absent in six, was present in four cases, and inconstant in one. Hence the sign, though not without value, is clearly not to be depended on. Secondary cancer is very rare; a case of secondary melanotic sarcoma, and another where a fragment of carcinoma of the œsophagus had become implanted on an old ulcer of the stomach have recently been reported.[2] Primary sarcoma is also rare, there being only about 60 cases on record. As the mucous membrane is often not involved till late in the disease, there may be nothing to lead the patient to suspect that he is ill for a long period. Soltau Fenwick[3] notices that six varieties of primary sarcoma have been found, viz., round celled, spindle celled, and myo-, myxo-, fibro-, and angio-sarcoma. The growths secondary to these appeared on the skin in 12 per cent. of the cases. S. S. Burt[4] recently met with a case of colloid cancer of the stomach and omentum. There had been slight pain and discomfort and ascites for several months, and, when the fluid was drawn off, some indefinite masses were felt in the left upper quadrant of the abdomen. By chance, too, some fragments of the growth came away with the fluid, which enabled a diagnosis to be made. The disease is exceedingly rare in this situation, and has probably never been diagnosed before during life except after an exploratory operation. The growth itself may easily be mistaken under the microscope for tubercle.

**Gastric Ulcer.**—The administration of opium and morphine is inadvisable unless absolutely necessary, for they increase the amount of H.Cl. excreted in many cases. Lepine[5] on this account advises that atropine or large doses of sodium bicarbonate should be given instead to relieve pain. Hirsch and Riegel too find that morphine causes pyloric spasm with primary lessening of the H.Cl. followed by a secondary increase. D. D. Stewart[6] in describing his plan of treatment advises rectal feeding for one to three weeks. The meals are given three times a day and do not exceed eight ounces, the rectum is washed out before each feeding, and if irritable has a small iodoform suppository inserted after the washing. Every third day the stomach is washed out with 500 c c. of a solution of silver nitrate (1 in 1,000) for about three minutes, then with several changes of plain water, and finally a teaspoonful of bismuth is introduced. On the intervening day a gramme of alumno is introduced with a teaspoonful of nitrate of bismuth in a pint of water, the latter being syphoned off. When hæmorrhage or vomiting is troublesome there is substituted a dose of a teaspoonful of bismuth three times a day instead of this local treatment, but later on the silver and alumnol douches are employed for one to three months at longer intervals. No food is given for an hour after bismuth. If hyperacidity is present, soda, magnesia, and calcium carbonate are given. Peptonised milk gruel is the first food given by the mouth followed by thickened broth, pulped meat, and eggs. Milk alone is objectionable, because it cannot be prevented from clotting. W. Calwell[7] records several cases of operation for perforation, and lays down that there is a definite *type of dyspepsia due to adhesions.* There may be a history of gastric ulcer or of localised pain and tenderness near the pylorus. The dyspeptic troubles disappear while the patient is kept in bed on easily digested food; the pain is of a dragging character increased by exertion, and becomes sharp and shooting upon lifting a weight or stretching. There is some relief from wearing a bandage. In many instances the patient is hungry but afraid to eat. For such cases after ordures inary meas he advises an operation, for the disease may disable a patient for years and end in perforation, but we must distinguish from them chronic dyspepsia due to bad food, hysteria, latent ulcer, neurasthenia and hyperchlorydria. W. R. Stokes[8] discusses an ulcer of the stomach caused by the diphtheria bacillus. It appears that Schoedel and others have noted cases of gastric diphtheria showing that the stomach is not always able to destroy the bacilli, especially when its secretions are weakened by an acute disease. The ulcer in this case was near the pylorus on the lowest part of the greater curvature, and consisted of a mass of coagulation necrosis which had replaced the entire mucous membrane and part of the submucosa. The advisability of surgical interference in various stomach lesions is discussed by Kauffman.[9] All are agreed as to the need of operation in the case of recent perforation by an ulcer or a foreign body, and in the case of simple strictures of the pylorus or cardia, or of cancerous strictures which are freely movable, but there is more doubt as to laparotomy for adhesions producing long continued dyspepsia and pain. These are difficult to locate and if on the posterior wall are most difficult to operate on. Moreover, in some of these cases the stomach will in time accommodate itself to the adhesion, especially if bulky food be avoided. It is also doubtful whether operation should be advised for ulcers which have not perforated but which threaten life by repeated bleeding. In supposed cancer without a palpable tumour but great and advancing anæmia, operation should be recommended, but if there is no marked anæmia there is great difficulty in diagnosing cancer from chronic dyspepsia. If there is a tumour which is not freely movable and the patient's condition is good, we may attempt resection or open the stomach into the bowel. In chronic dilatation and failure of motor power dependent on deficient muscular power no operation is desirable until all other methods have been long and carefully tried. Perigastric adhesions, according to Bird,[10] may be due to ulcers, to affections of the bile ducts, to syphilis, trauma, and other causes. Among their symptoms is pain definitely localised, coming on half an hour after food, often relieved by the recumbent posture, aggravated or aroused by movement, and varying in intensity from a dull ache to extreme agony. Vomiting is not a prominent symptom. A form of gastric pain due to neuralgia is discussed by T. S. Short.[11] The diagnosis is certainly difficult, for we must eliminate organic disease, especially ulcer, cancer, and dilatation. In simple neuralgia the attacks may occur daily and sometimes at a fixed hour, food has no effect and the nutrition of the body is good. Recurring pains may be due to gall stone colic, but here the attacks are not so frequent; aneurism, lead colic, angina, and tabetic crises must be also excluded; and even cases of poisoning, movable kidney, and the irritation due to the presence of worms may give rise to very similar symptoms.

[1] Med. Rec., April 27. [2] Practitioner, June, p. 686. [3] Practitioner, June, p. 685. [4] Med. Rec., July 6. [5] Brit. Med. Jour., March 23. [6] Quarterly Med. Jour., August. [7] Dublin Jour. Med. Sc., August. [8] Johns Hopkins' Bulletin, July. [9] Birm. Med. Rev., August. [10] Amer. Jour. Med. Sc., July. [11] Birm. Med. Rev., April.

